# Effects of COVID-19 lockdown on university students’ anxiety disorder in Italy

**DOI:** 10.1186/s41118-021-00135-5

**Published:** 2021-10-09

**Authors:** Giovanni Busetta, Maria Gabriella Campolo, Fabio Fiorillo, Laura Pagani, Demetrio Panarello, Valeria Augello

**Affiliations:** 1 Department of Economics, University of Messina, Via Dei Verdi 75, 98122 Messina, Italy; 2 Dipartimento di Scienze Economiche e Sociali, Università Politecnica delle Marche, Ancona, Italy; 3 Department of Economics and Statistics, University of Udine, Udine, Italy; 4 Department of Statistical Sciences “Paolo Fortunati”, University of Bologna, Via delle Belle Arti 41, 40126 Bologna, Italy; 5 S.A.M.O ONLUS , Via Giusti 33, Palermo, Italy

**Keywords:** STAI test, Coronavirus outbreak, Pandemic, Mental health, State and trait anxiety

## Abstract

The COVID-19 pandemic has highlighted the vulnerability of specific population sections, with regards to economic and work conditions, mental and physical well-being, and context-based factors, emphasizing the need for timely policy measures aimed at counteracting the Italian economic framework’s fragility—which poorly adapts to unexpected circumstances. Identifying the most vulnerable groups is, therefore, essential with a view to carrying out targeted measures. Concerning University, the economic downturn caused by COVID-19 could likely result in a decrease in enrollments to both the first and further years of study, with significant consequences on the future of students and the system as a whole. The class of students is of great interest, as it is made up of individuals differing from each other in many ways. Our investigation is aimed at observing anxiety levels filtering the perception of one’s anxiety state in a highly stressful time such as the pandemic from the usual anxiety levels. This evaluation allows us to evaluate the similarity of individual behaviors during the lockdown period with those from the previous period.

## Introduction

The rapid spread of COVID-19 has turned into a global public health crisis. The COVID-19 pandemic produced negative psychological problems other than mortality, including increased anxiety (see Fardin, 2020 for an overview of this strand of literature).

Psychological literature predicts an increase in anxiety-related disorders, during stressful periods such as pandemics for the prevalence of the connected increase in stress, anxiety and depression (Salari et al., 2020). However, different groups may have different reactions, also depending on their initial anxiety level (Holmes et al., 2020). Responses to past pandemics are instructive to evaluate the reaction of individuals in terms of health anxiety (McKay, 2020). During the H1N1/09 pandemic, the level of anxiety reached the highest point at the peak of the pandemic and decreased right away (Salari et al., 2020). Similar results were revealed during the Ebola pandemic by Olatunji et al. (2007) and during the Zika outbreak in 2015–16 by Blakey & Abramowitz (2017).

The nature of COVID-19 implies an even more prominent correlation between fear of contracting the virus and increase in the level of health anxiety, due to the connected particularly relevant respiratory symptoms, which is generally associated by the literature to anxiety sensitivity (see Horenstein et al., 2018 for a review of this literature).

Moreover, individuals characterized by a primary current anxiety-related disorder are more affected by the pandemic, compared to those affected by other or no mental disorders (Asmundson et al., 2020). Deeply, during the COVID-19 pandemic, empirical evidence shows widespread emotional distress in China (Qiu et al., 2020; Shigemura et al., 2020; Wang et al., 2020a, 2020b), Japan (Ueda et al., 2020), UK (Shevlin et al., 2020), Spain (Odriozola-González, 2020), and Italy (Mazza, 2020).

On the one side, the increase in the level of anxiety during a pandemic could relate, among other things, to the increase in danger and contamination fears (Taylor et al., 2020a, 2020b) and to the anxiety, depression and insecurity (Salari et al., 2020) connected to the pandemic. On the other side, it could relate to the disruptions of routines and mental health care, caused by conditions of social isolation, such as in the case of lockdown or quarantine (Chatterjee et al., 2020; Druss, 2020; Salari et al., 2020; Yao et al., 2020). During the SARS outbreak in 2003, Hawryluck et al. (2004) showed a significant correlation between the duration of quarantine and the prevalence of post-traumatic stress disorder (PTSD) symptoms.

The continuous spread of the pandemic, strict isolation measures and delays in starting schools, colleges, and universities across the country is expected to influence students’ mental health. There have been reports on the psychological impact of the pandemic on the public, patients, medical staff, children, and older adults (Chen et al., 2020; Li et al., 2020; Yang et al., 2020).

Concerning University, the economic downturn caused by COVID-19 could likely result in a decrease in enrollments to both the first and further years of study, with significant consequences on the future of students and the system as a whole. The class of students is of great interest, as it is made up of individuals differing from each other in many ways. Suffice it to think about differences between on-site and off-site students, or between full-time and working students. Furthermore, according to the main literature (see Salari et al., 2020 for an overview), greater levels of anxiety, depression, and stress commonly affect people with higher levels of education and this correlation makes university students a particularly relevant sample to evaluate the impact of the current pandemic on anxiety levels.

In December 2019, the Novel Coronavirus SARS-CoV-2 was first detected in Wuhan, China. About 1 month later (January the 31st), Italy joined the list of coronavirus-affected countries due to two Chinese tourists confirmed to be infected by COVID-19. A third COVID-19 case was confirmed on February the 7th; the number of confirmed infections furtherly increased on February the 21st, with sixteen new cases found in the regions of Lombardy and Veneto. Following the first two COVID-19-related deaths, several towns in Lombardy were locked down due to the large number of infected patients in the area. Italy implemented a nationwide lockdown on the 10th of March, soon followed by most countries worldwide, to contain and curb the transmission of the disease (Panarello & Tassinari, 2021). As a consequence, non-essential commercial and industrial activities were closed, sporting and cultural events and religious activities were suspended, and the shutdown of schools and universities was introduced in the whole country. Italian university students began to experience a new kind of university life, characterized by social isolation, distance learning, and online relationships with their peers. The lockdown came to an end on the 4th of May. The restrictions were loosened until the end of September: freedom of movement across Italy and other European countries were authorized, and schools and Universities were open. Further restrictive measures were then introduced due to a new “wave” of COVID-19. Starting from November 2020, Italy was divided into three “colored” zones (red, orange, yellow), characterized by different restrictive measures on the basis of the severity of the spread of COVID-19 at the regional level. Schools and universities were ordered to close in the regions with greater pandemic severity. The vaccination campaign started at the end of December 2020; then, since February 2021, Italy suffered from a third “wave” of COVID-19.^1^ The decrease in deaths and infections since April 2021, combined with a massive vaccination campaign, allowed to reduce the restrictions’ severity. In July, the EU Digital COVID Certificate was introduced and made compulsory to access some categories of public places. Since the 1st of September, university students and academic personnel are only allowed to access university if in possession of the COVID certificate.

Following Amerio et al. (2021), during the national lockdown in Italy, the national-level prevalence of depressive and anxiety symptoms doubled. Moreover, several analyses revealed high rates of negative mental health outcomes in the Italian general population caused by COVID-19 lockdown measures (Rossi et al., 2020).

Specifically, young and more educated subjects are more likely to present a worsening in depressive and anxiety symptoms during the COVID-19 pandemic, compared to older people (Amerio et al., 2021). The reduced ability of the youth to tolerate uncertainty about the future could explain these differences in terms of mental health reactions depending on the age of the subject (Ettman et al., 2020).

Several analyses applied to university students showed higher rates of anxiety/depression and financial instability due to the pandemic all over the world (e.g., Esteves et al., 2021; Garvey et al., 2021; Jones et al., 2021). In this respect, Jones et al. (2021), studying the City University of New York, underlined that more than half of the interviewed students (54.5%) reported relevant levels of anxiety/depression and an increased need for mental health services due to the pandemic. Esteves et al. (2021) detected a positive association between high levels of anxiety symptoms and occurrence of sleep problems among Brazilian university students during the lockdown period. Garvey et al. (2021), using the GAD-7 (7-items Generalized Anxiety Disorder scale) test to detect anxiety levels in Spain, found a relevant proportion of university students affected by high levels of anxiety connected to the pandemic.

Concerning Italian university students, Fornili et al. (2021) show a connection between severe levels of anxiety and low levels of income, irrespective of their health conditions and worries about contracting the virus.

Even if strict isolation measures connected to the widespread diffusion of COVID-19 imply deep psychological consequences on students attending schools, colleges and universities, no detailed studies have been conducted on the specific impact on Italian university students.

Therefore, this study focuses on the psychological impact of the COVID-19 outbreak among Italian university students.

The current research aims at exploring anxiety levels among students of three Italian Universities, respectively, located in the South, Centre and North of Italy, during the lockdown.

According to the literature (see Rajkumar, 2020 for a review), supplies and family affluence are negatively correlated with depression, stress and anxiety (Jewell et al., 2020). Deeply, a greater psychological impact of the outbreak and higher levels of anxiety and depression are significantly associated with female gender, students’ age, income, and living together with parents (Aylie et al., 2020).

We want to test the following hypotheses:

- H1: State anxiety during the lockdown is higher than trait anxiety.
- H2: Differences in the development of state anxiety can be observed depending on students’ initial level of anxiety.
- H3: Students’ economic situation can impact anxiety levels.
- H4: Women are more likely to develop a high level of state anxiety compared to men.

### Survey

For our analyses, we use data from an ad hoc questionnaire, administered during the lockdown to the students of three Italian universities: Messina (southern Italy), Udine (northern Italy), and the Marche Polytechnic (central Italy) in Ancona. The universities, classified as medium-sized, are mainly attended by students living in neighboring areas, and located in three different Italian macro-regions (North, Center and South) characterized by very different socio-economic conditions. Therefore, the idea is that students attending these universities are homogenous with respect to their “local” lifestyle. Moreover, none of the three cities has experienced very high COVID-19 contagion rates. The gathered evidence concerns demographic, economic, labor, context-based, online teaching, time use, and psychological well-being features. All the students from all departments of the three universities were invited to take part in the survey, which was open from the 29th of April to the 17th of May 2020. We chose to submit the questionnaires in this period as it was in full pandemic and quite far from exam sessions (so to avoid the quote of anxiety that could derive from the exams’ period). The distribution of some students’ characteristics such as sex, university degree and area of study, in the population (P) and in the sample (S) of respondents, together with the participation rate, are reported in Table 1. The data refer to the academic years 2019–2020.

**Table 1:** Population (P) and sample (S) characteristics

	Messina		Marche Polytechnic		Udine		All	
	P	S	P	S	P	S	P	S
	%	%	%	%	%	%	%	%
Sex								
Male	37.0	27.0	55.0	44.0	48.0	29.0	45.0	34.0
Female	63.0	73.0	45.0	56.0	52.0	71.0	55.0	66.0
Degree								
Bachelor’s degree	60.1	63.2	65.9	66.0	68.0	61.0	64.0	63.7
Master’s degree	14.5	16.5	20.3	23.0	16.0	21.8	16.6	20.5
Single-cycle master program	21.7	19.3	10.6	10.7	13.4	13.1	16.2	14.2
Specializing master or specialization degree	3.6	0.7	3.1	0.0	2.5	1.5	3.1	0.6
PhD	0.1	0.3	0.1	0.3	0.1	2.6	0.1	1.0
Area of study								
Economics and law	17.9	17.5	9.8	16.2	17.8	19.8	15.4	17.6
Medicine	28.5	24.1	20.7	19.2	10.5	11.5	21.1	18.7
Sciences	20.5	28.1	57.3	64.6	42.1	36.6	37.7	44.6
Humanities and education	33.1	30.3	12.2	0.0	29.6	32.1	25.8	19.1
Overall								
Percentage	42.7	33.4	28.9	38.6	28.4	28.0	100.0	100.0
Size	22,346	1461	15,095	1690	14,959	1228	52,490	4379
Participation rate	6.5		6.5		11.2		8.2	

The total number of answers to the survey was: 1228 from the students at the University of Udine (28%), 1461 from the University of Messina (33%), and 1690 from the Marche Polytechnic University (39%): in sum, 4379 answers were gathered in 19 days.

The sample includes 2868 females (66%) and 1,511 males (34%). Most students are enrolled in a bachelor’s degree course (2789, 64%). Regarding the field of studies, we divided the different disciplines into four major groups (Economics and Law; Medicine; Sciences; Humanities and Education). 1953 students are enrolled in a science course (45%), 836 in Humanities and Education (19%), 817 in Medicine (19%), and 773 in Economics and Law (18%).

The percentage of students involved in the survey who filled the questionnaire is 8.3%, ranging from 6.5% (University of Messina) to 11.2% (Marche Polytechnic University).

### Measures

Deeply, this paper focuses on anxiety, which is commonly defined as an adaptive emotion, preparing individuals to identify and face threats to guarantee their own survival. Deeply, anxiety is defined as the anticipation of a future threat, frequently associated with the muscular tension of alertness in response to future danger and to prudent or avoidance behavior (American Psychiatric Association, 2013).

Cattell (1966) differentiates anxiety as an emotional state and anxiety as a personality trait. The STAI-Y (State-Trait Anxiety Inventory) test (Julian, 2011), included in the survey, is a self-report test to measure the presence and the level of anxiety. It is composed of two subscales: the State Anxiety Scale (S‐Anxiety), which evaluates the current state of anxiety with items asking to respond to how people feel “right now”; and the Trait Anxiety Scale (T‐Anxiety), which evaluates a stable measure of anxiety.

Using this test, the investigation allows us to observe the variation in anxiety levels, filtering the perception of one’s anxiety state in a highly stressful time, such as the pandemic from the anxiety level as a personality trait.

Deeply, we administered the STAI-Y test to university students to separate state anxiety (in our case, the one concerning the lockdown period) and trait anxiety (the level of anxiety as a personal characteristic).

The STAI is composed of 40 items: 20 items for each subscale. Each scale consists of 20 items that are rated on a 4-point Likert scale. The S‐Anxiety items evaluate current feelings “at this moment”: (1) not at all, (2) somewhat, (3) moderately so, and (4) very much so. The T‐Anxiety scale items evaluate the frequency of feelings “in general”: (1) almost never, (2) sometimes, (3) often, and (4) almost always. The original STAI‐X was first published in 1970, then revised in 1983 as the STAI‐Y, which has been used extensively. The STAI-Y is a self‐report questionnaire in an individual format, and it takes about 10 min for adults to complete. For each subscale, specific instructions are provided. Each subtest (state and trait) has a range of scores from 20 to 80, the higher score indicating a higher level of anxiety. To detect clinically significant symptoms for the S‐Anxiety scale, a cut point of 39–40 has been suggested (Addolorato et al., 1999; Knight et al., 1983); moreover, three levels of anxiety can be detected, divided into low (from 20 to 37), moderate (from 38 to 44), and high (from 45 to 80). Normative values are available in the manual (Avery et al., 1990) for adults, students, and psychiatric samples.

The evaluation of structural breaks in the temporal dynamics of the analyzed phenomena and the analysis of the unobserved aspects of personality and well-being traits allow us to evaluate whether individual behaviors during the lockdown period have changed compared to the habitual ones.

## Methods

For our analyses, we estimate two Probit Models (Models A and B). We aim at investigating the potential transition to a state anxiety level that is higher than trait anxiety, creating two dichotomous variables to be used as dependent.

In Model A, the dependent variable takes value 1 if the State anxiety level is high and 0 otherwise.

In Model B, the dependent variable takes value 1 for students who are in a high State anxiety level (i.e., from 45 to 80) and with a state anxiety score higher than the trait anxiety score.

To better understand the impact of the employed variables on the outcome, we also compute the marginal effects of the two Probit regressions. The parameter estimates from the probit model must be transformed to yield estimates of the marginal effects, i.e., the change in predicted probability associated with changes in the explanatory variables (see Greene, 2012). Marginal effects are nonlinear functions of the parameter estimates and the levels of the explanatory variables, so they cannot generally be inferred directly from the parameter estimates (Anderson & Newell, 2003). To compute standard errors, we can use the linear approximation approach (delta method, see Greene, 2012, p. 733). For factor levels, the marginal effect is the discrete change from the base level. In particular, in the case of dummy variables, the marginal effect is simply the difference in probability when the indicator variable *X* changes from 0 to 1, given by:

$$E\left( {y|x1 = 1} \right) - E\left( {y|x1 = 0} \right).$$

Moreover, it is interesting to consider the way in which students with low or moderate trait anxiety worsen their level of anxiety during the lockdown and the way in which students with high anxiety do, but also whether there are some students with high trait anxiety which are better off during the lockdown. Therefore, we build matrixes of transition from trait level to state level.

### Descriptive results

In Table 2, we show the descriptive statistics of the sample by anxiety level.

**Table 2:** Characteristics of the survey respondents by anxiety level

	Overall	State			Trait		
		Low (*n* = 564)	Moderate (*n* = 646)	High (*n* = 3169)	Low (*n* = 886)	Moderate (*n* = 975)	High (*n* = 2518)
Sex							
Male	0.35	0.21	0.20	0.60	0.27	0.24	0.48
Female	0.65	0.09	0.12	0.79	0.17	0.21	0.62
Age							
18–21	0.42	0.13	0.15	0.71	0.20	0.23	0.57
22–25	0.38	0.11	0.14	0.75	0.19	0.22	0.59
26–30	0.14	0.13	0.14	0.73	0.20	0.20	0.59
> 30	0.06	0.20	0.18	0.62	0.27	0.23	0.50
University							
Messina	0.33	0.10	0.14	0.76	0.18	0.21	0.60
Marche	0.39	0.15	0.16	0.69	0.21	0.23	0.55
Udine	0.28	0.13	0.13	0.73	0.21	0.22	0.57
Degree							
Bachelor’s degree	0.64	0.13	0.14	0.73	0.19	0.21	0.59
Master’s degree	0.21	0.13	0.14	0.73	0.23	0.22	0.55
Single-cycle master program	0.14	0.14	0.18	0.68	0.20	0.26	0.54
Specializing master or specialization degree	0.01	0.18	0.07	0.75	0.29	0.21	0.50
PhD	0.01	0.12	0.10	0.79	0.24	0.31	0.45
Area of study							
Economics and law	0.18	0.10	0.14	0.76	0.19	0.23	0.58
Medicine	0.19	0.15	0.17	0.68	0.22	0.23	0.55
Sciences	0.45	0.14	0.16	0.71	0.21	0.23	0.56
Humanities and education	0.19	0.11	0.11	0.78	0.19	0.19	0.62

In general, students report high levels of anxiety. About 57.5% of the sample shows a high level of trait anxiety.

The mean state anxiety score was 52.7, with a standard deviation of 12.24. On the other hand, the mean trait anxiety score was 47.3, with a standard deviation of 11.11. This result shows that students’ average level of anxiety and its standard deviation increased during the lockdown with respect to the habitual levels. Although emotional spectrum disorders can be influenced by seasonal characteristics, in the DSM-5 (American Psychiatric Association, 2013), among the diagnostic criteria of anxiety disorders, in the risk and prognosis factors and in the specifiers, the seasonal element is never present as a factor that significantly affects the anxiety disorder (whatever type it is within all anxiety disorders). Therefore, the restrictive measures such as university closures led to negative emotions in the students’ population on average (H1).

The difference between state and trait is significant in the three universities, with a higher level of state score compared to trait score (see Table 3).

**Table 3:** Trait and state anxiety scores by university

University	State		Trait		Diff	*t*	*p* value
	Mean	Std. Err	Mean	Std. Err			
Messina	54.11	0.31	48.11	0.30	6.00	13.93	***
Marche	51.32	0.30	46.54	0.26	4.79	12.02	***
Udine	52.91	0.35	47.48	0.32	5.43	11.41	***

***p value < 0.0001

Table 4 shows the matrix of transition from trait to state levels of anxiety. Indeed, the resulting groups should be considered as different ones in terms of policy interventions to alleviate their anxiety problem. Concerning the group of students who do not suffer from anxiety and start suffering because of the pandemic shock, a policy aimed at reducing the reasons for which they suffer from the impact of the shock could also solve their anxiety problems. Another group is composed of fragile students with anxiety problems: in this case, we could venture that, for high initial levels of anxiety, the increase in anxiety levels is induced by the fear of contagion during a lockdown (Horenstein et al., 2018). This group suffers from pathological anxiety and should, therefore, be protected the most from the lockdown/pandemic effect.

**Table 4:** Transition matrix by level of trait and state anxiety

Trait level	State level			
	Low	Moderate	High	Total
State score > = Trait score				
Low	222	205	347	774
Moderate		131	625	756
High			1600	1600
Total	222	336	2572	3130
State score < Trait score				
Low	112			112
Moderate	130	89		219
High	100	221	597	918
Total	342	310	597	1249

We note that, among students with a low trait anxiety level (774 + 112 = 886, also see Table 2), 347 (39.16%) start presenting a high level of anxiety during the lockdown. Among the 975 students affected by a moderate trait anxiety level, 625 (64.10%) transit to a high state anxiety level. Thus, out of the 1861 students who did not suffer from relevant anxiety problems, 972 (52.23%) start experiencing such issues.

Moreover, we note that out of the 2518 fragile students (i.e., those who suffer from a high level of trait anxiety), 1600 (63.54%) have worsened their problem during the pandemic. For these students, probably, more intense interventions could be necessary with a view to protecting them.

It is noteworthy that 918 students with high trait anxiety are better off (36.46%) during the pandemic, and, among them, 321 students (100 + 221) seem to have relevantly reduced their anxiety problems by transiting to a lower level of anxiety. This suggests that, during the lockdown, there are some elements that can reduce the state level of anxiety. We can argue that this is due to the change in lifestyle, suggesting that further investigations are needed to identify which lifestyle elements could have such a positive impact.

Differences in the development of state anxiety can be noticed depending on students’ initial level of anxiety (Table 4), confirming the H2 hypothesis.

Indeed, while people who are usually not anxious can experiment a high increase in anxiety due to the pandemic fear, people who are usually very anxious can experiment lower pace of life due to the confinement measures, which may lead to a lower increase in their level of anxiety. The increase in anxiety is always higher in Messina, albeit the rates of contagion in the region had always been lower than in central and northern Italian regions.

In the following table (Table 5), we report the composition of the sample by trait and state anxiety level, based on the previous transition matrix (Table 4).

**Table 5:** Composition of the sample by trait and state anxiety

Trait	State score ≥ Trait score			State score < Trait score		
	Low	Moderate	High	High	High	High
State	High	High	High	High	Moderate	Low
	(*n* = 347)	(*n* = 625)	(*n* = 1600)	(*n* = 597)	(*n* = 221)	(*n* = 100)
Sex						
Female	0.70	0.68	0.74	0.71	0.56	0.58
Male	0.30	0.32	0.26	0.29	0.44	0.42
Age						
18–21	0.39	0.42	0.42	0.42	0.40	0.40
22–25	0.40	0.40	0.39	0.40	0.39	0.34
26–30	0.15	0.12	0.14	0.13	0.16	0.13
> 30	0.06	0.05	0.05	0.04	0.05	0.13
University						
Messina	0.31	0.32	0.37	0.35	0.29	0.18
Marche	0.36	0.39	0.36	0.38	0.45	0.42
Udine	0.33	0.29	0.27	0.28	0.27	0.40
Degree						
Master’s degree	0.24	0.21	0.22	0.17	0.17	0.16
Single-cycle master program	0.13	0.16	0.13	0.13	0.20	0.15
Bachelor’s degree	0.60	0.61	0.65	0.69	0.63	0.67
Specializing master or specialization degree	0.01	0.00	0.00	0.01	0.00	0.01
PhD	0.02	0.01	0.01	0.01	0.00	0.01
Area of Study						
Economics and law	0.17	0.19	0.21	0.14	0.13	0.09
Medicine	0.18	0.16	0.18	0.17	0.20	0.20
Sciences	0.45	0.46	0.40	0.49	0.51	0.54
Humanities and education	0.20	0.18	0.22	0.20	0.16	0.17

To better test this study’s hypotheses, we perform a *t* test on the difference in means for all covariates used in our model, considering trait and state anxiety scores separately (Table 6).

**Table 6:** Difference in means for the employed variables (*t* test)—state and trait anxiety

	Obs	State				Trait			
		Mean	Diff	Std. Err	*p* value	Mean	Diff	Std. Err	*p* value
Female	2868	54.88	− 6.33	0.377	***	48.72	− 4.05	0.348	***
Male	1511	48.55				44.68			
Age 18–21	1851	52.39	0.53	0.374		47.37	− 0.07	0.340	
Age > 22	2528	52.92				47.29			
Messina	1461	54.11	− 2.12	0.391	***	48.11	− 1.18	0.355	**
Other	2918	51.99				46.93			
Marche	1690	51.32	2.24	0.379	***	46.54	1.29	0.344	***
Other	2689	53.56				47.82			
Udine	1228	52.91	− 0.29	0.412		47.48	− 0.21	0.374	
Other	3151	52.61				47.27			
Economics and Law	773	54.52	− 2.22	0.484	***	47.41	− 0.10	0.441	
Other	3606	52.30				47.31			
Medicine	817	51.65	1.28	0.474	**	46.72	0.75	0.431	
Other	3562	52.93				47.47			
Sciences	1953	51.62	1.94	0.371	***	46.98	0.62	0.338	
Other	2426	53.56				47.60			
Humanities and Education	836	54.54	− 2.28	0.469	***	48.65	− 1.63	0.426	***
Other	3543	52.26				47.01			
Bachelor’s Degree	2789	52.94	− 0.67	0.385		47.80	− 1.29	0.348	***
Other	1590	52.27				46.50			
Changed home	996	54.68	− 2.57	0.440	***	47.81	− 0.62	0.401	
Did not change home	3383	52.11				47.18			
Scholarship	1645	53.77	− 1.72	0.381	***	48.00	− 1.08	0.346	**
No scholarship	2734	52.05				46.92			
Outdoor space: garden or terrace	3428	52.04	3.00	0.447	***	46.88	2.04	0.406	***
No outdoor space	951	55.05				48.92			
Distance learning	3544	52.41	1.51	0.470	**	46.94	2.01	0.426	***
No distance learning	835	53.92				48.96			

*p < 0.05; **p < 0.01; ***p < 0.001

The H3 hypothesis is confirmed, showing that anxiety scores are higher for those with a scholarship. The H4 hypothesis is also confirmed, showing that anxiety scores are higher among women, with statistically significant differences for both trait and state scores.

It is interesting to investigate if students’ characteristics affect the average trait and state anxiety scores. In general, the average state score is higher than the trait score.

The characteristics that show a significant difference in the average state scores are: sex, living in a house with a garden or a terrace (Outdoor space), studying in Messina or Marche Polytechnic University, all the areas of study, having moved to a different home, receiving a scholarship and attending online lessons.

Considering the average trait scores, the characteristics that show a significant difference are: sex, living in a house with a garden or a terrace (Outdoor space), studying in Messina or Marche Polytechnic University, attending a course in the Humanities and Education area of studies, the degree level, receiving a scholarship and attending online lessons.

### Probit analysis

As revealed by the descriptive analyses, it is crucial to focus on those students who show a pathological level of anxiety (STAI state score from 45 to 80), being the ones who should be protected the most from the lockdown/pandemic effect. For our analyses, we estimate two Probit Models (Models A and B). We aim at investigating the potential determinants of a high level of state anxiety, creating two dichotomous variables to be used as dependent.

In Model A, the dependent variable takes value 1 if the State anxiety level is high and 0 otherwise. In Model B, the dependent variable takes value 1 for students who are in a high State anxiety level and with a state anxiety score higher than the trait anxiety score.

In the following tables, we show the results of the two Probit models (Table 7) and their marginal effects (Table 8).

**Table 7:** Estimation results of the Probit models

	Model A			Model B		
	Coef	Std. Err	*p* value	Coef	Std. Err	*p* value
Dummy sex: 1 = female	0.584	0.045	***	0.385	0.043	***
Dummy age: 1 = 18–21	− 0.079	0.048		− 0.045	0.045	
University (Ref: Messina)						
Marche polytechnic	− 0.139	0.055	*	− 0.072	0.051	
Udine	− 0.045	0.056		− 0.017	0.052	
Dummy course degree: 1 = Bachelor’s	0.107	0.048	*	− 0.006	0.045	
Area of study (Ref: Economics and Law)						
Medicine	− 0.308	0.069	***	− 0.321	0.065	***
Sciences	− 0.060	0.061		− 0.196	0.056	**
Humanities and education	− 0.135	0.073		− 0.157	0.067	*
Dummy changed home: 1 = Yes	0.232	0.051	***	0.266	0.047	***
Dummy scholarship: 1 = Yes	0.094	0.044	*	0.085	0.040	*
Dummy outdoor space: 1 = Yes	− 0.234	0.055	***	− 0.141	0.049	**
Dummy distance learning: 1 = Yes	− 0.148	0.056	**	− 0.053	0.051	
Constant	0.600	0.093	***	0.213	0.086	*

*p < 0.05; **p < 0.01; ***p < 0.001

**Table 8:** Marginal effects of the Probit models

	Model A			Model B		
	dy/dx	Std. Err	*p* value	dy/dx	Std. Err	*p* value
Dummy sex: 1 = female	0.197	0.015	***	0.150	0.016	***
Dummy age: 1 = 18–21	− 0.025	0.015		− 0.017	0.017	
University (Ref: Messina)						
Marche polytechnic	− 0.044	0.017	*	− 0.027	0.020	
Udine	− 0.014	0.017		− 0.006	0.020	
Dummy course degree: 1 = Bachelor’s	0.034	0.015	*	− 0.002	0.017	
Area of study (Ref: Economics and Law)						
Medicine	− 0.099	0.022	***	− 0.122	0.024	***
Sciences	− 0.018	0.018		− 0.074	0.021	***
Humanities and education	− 0.042	0.022		− 0.059	0.025	*
Dummy changed home: 1 = Yes	0.071	0.015	***	0.100	0.017	***
Dummy scholarship: 1 = Yes	0.030	0.014	*	0.032	0.015	*
Dummy outdoor space: 1 = Yes	− 0.071	0.016	***	− 0.054	0.019	**
Dummy distance learning: 1 = Yes	− 0.045	0.017	**	− 0.020	0.019	

*p < 0.05; **p < 0.01; ***p < 0.001. dy/dx for factor levels is the discrete change from the base level

The first model (Model A) estimates the probability of being in a pathological level of state anxiety. The results roughly confirm those from the descriptive analyses and tests.

The second model (Model B) investigates the probability of increasing the pathological anxiety situation (i.e., the probability to be usually in a non-pathological state but to move into a pathological one during the lockdown, or to start from a pathological situation worsening it even more). It is more likely to be in such a state for women, for those receiving a grant due to income reasons, and for those who changed their homes during the lockdown. The probability to be in this anxiety state decreases for students attending university courses in Medicine, Sciences, and Humanities and Education (compared to Economics and Law) and for those who benefit from outdoor facilities, such as a garden.

The marginal effects (Table 8) show that women, compared to men, are almost 20% more likely to experience a high level of state anxiety, which decreases to 15% for fragile students (high level of state anxiety with state score higher than trait score). University students from medical areas, compared to the reference group, are about 10% less likely to experience a high level of state anxiety (over 12% for the fragile group), probably due to being more informed about the way the disease works and its connected consequences. Compared to students who did not change their living habits, those who changed their home during the pandemic are statistically more likely to experience a high level of anxiety (7% and 10% for the two groups), and the same goes for students who benefit from a scholarship (about 3% for both groups). Students who benefit from outdoor spaces (such as a garden or a terrace) are less likely to show high anxiety levels (7% and 5% for the two groups). Finally, we can observe that distance learning has a beneficial effect on students’ level of anxiety, but only for the first group (− 4.5%).

**Fig. 1:**
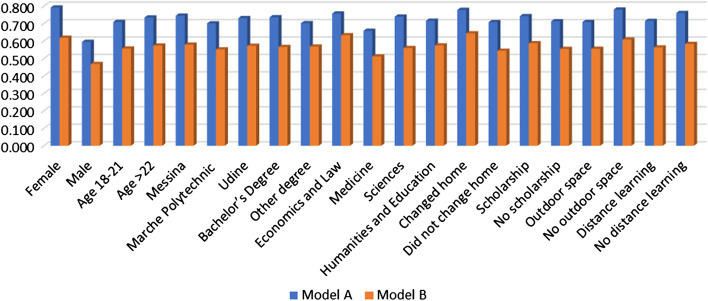
Predicted probabilities for the Probit models

Finally, in the following graph, we show the predicted probabilities for the two estimated Probit models (Fig. 1).

## Conclusions

The pandemic condition produces several problems, changing health perceptions and worsening students’ expectations about the future. Due to the COVID-19-related restrictions, students have experienced a new way to approach university life, with social distancing and remote learning (online lessons and exams), with a deep impact on their social life and mental health.

Pathological anxiety among students is a problem that needs to be faced. Our study investigates the impact of the COVID-19 outbreak on anxiety among Italian university students located in the South, Centre, and North of Italy, during the lockdown. As the seasonal element is never present in the DSM-5 as a factor that influences the anxiety disorder in a significant way (American Psychiatric Association, 2013) and the period chosen for submitting the questionnaire is sufficiently far from the exam sessions, the only possible reason for this increase in anxiety level must be connected to the pandemic.

Our four hypotheses are confirmed by the performed analyses. As we could expect from the literature, state anxiety during the lockdown is higher than trait anxiety (H1). More than 50% of our sample starts suffering from anxiety during the lockdown and more than 60% of students who are affected by a high level of trait anxiety have worsened their problem during the pandemic.

These fragile students need more intense interventions to protect them from anxiety problems. It is worth noting that, among students with high trait anxiety, about 36% reaches a lower level of anxiety, maybe due to the change in their daily routine, which suggests that further investigations are needed to identify which lifestyle elements could have a positive impact. Such investigation may provide university authorities with some useful elements to improve students’ psychological health.

The investigation provides useful insights into the factors which may determine students’ level of state anxiety and its worsening compared to trait anxiety.

In addition, women show greater anxiety levels than men, confirming H4. The probability of increasing the pathological anxiety situation decreases for students attending university courses in Medicine, Sciences, and Humanities and Education. The same is true for house facilities. On the contrary, students who changed their house experienced a worsening in anxiety. As we expect (H3), a low economic condition (proxied by study grants received due to income conditions) is associated with higher state anxiety compared to trait anxiety.

As Italian universities have made a major effort to avoid interrupting academic activities, we also investigate the effect of online lectures on anxiety. We observed that distance learning has a beneficial effect on students’ level of anxiety, but not for the most vulnerable ones.

The necessity of gathering data within a short timeframe at the beginning of the pandemic forced us to administer our survey only to students enrolled in three Italian universities. Despite this, our results highlight some of the challenges faced by the university population during the pandemic. Further investigation is needed to consider the effect of the pandemic shock on the mental health of young populations. It would be necessary to consider other methods to investigate further mental conditions, such as depression and panic. Moreover, it is crucial to identify the characteristics of fragile and resilient subjects, with a view to facing unpredictable negative shocks in case they occur again, even though trait anxiety in the students’ population appears to be worryingly high, as evidenced in over 57% of the analyzed sample.

## Data Availability

The authors will share the data upon request.

## Footnotes

1 The full list of decrees is available at https://www.governo.it/it/coronavirus-misure-del-governo.
